# Spatial and spatiotemporal machine learning models for COVID-19 dynamics: a review of methodology and reporting practices

**DOI:** 10.1093/epirev/mxaf017

**Published:** 2025-10-23

**Authors:** Hassan K Ajulo, Faith O Alele, Theophilus I Emeto, Oyelola A Adegboye

**Affiliations:** Public Health and Tropical Medicine, James Cook University, Townsville, QLD, Australia; School of Health, University of the Sunshine Coast, Sippy Downs, QLD, Australia; Public Health and Tropical Medicine, James Cook University, Townsville, QLD, Australia; Public Health and Tropical Medicine, James Cook University, Townsville, QLD, Australia; Menzies School of Health Research, Charles Darwin University, Darwin, NT, Australia

**Keywords:** spatial, spatiotemporal, machine learning, coronavirus, COVID-19, infectious disease, epidemiology, public health

## Abstract

COVID-19 has transitioned from a pandemic to an endemic state, but the emergence of novel variants continues to pose significant public health challenges. In this study, the application of spatial and spatiotemporal machine learning (ML) models in understanding the dynamics of COVID-19 was systematically reviewed, as were contextual local-level comprehensive socio-environmental drivers. A systematic search was conducted across the Scopus, Web of Science, PubMed, Emcare (via Ovid), and the World Health Organization COVID-19 databases, and gray literature, adhering to Preferred Reporting Items for Systematic Reviews and Meta-Analyses guidelines. Data extraction was conducted according to the Critical Appraisal and Data Extraction for Systematic Reviews of Prediction Modeling Studies checklist, and study quality was assessed using a validated scoring system. A total of 42 studies met the inclusion criteria. The review Findings indicate that global-scale spatial and spatiotemporal ML models dominate the field. Long-standing standalone factors in the demographic, environmental, and socioeconomic domains are frequently used as local-level drivers. However, the integration of composite indicators, aggregating multiple standalone factors into a single score, is notably lacking. Such composite indicators have the potential to reduce model complexity, improve interpretability, and enhance performance by capturing multidimensional aspects of vulnerability or risk in a more simplified form. This review highlights critical gaps in the current use of spatial and spatiotemporal ML models to understand the spatial epidemiology of COVID-19. Addressing these gaps could significantly enhance the understanding of COVID-19 dynamics and inform the development of effective public health strategies to mitigate future threats.

## Introduction

The COVID-19 pandemic has exerted an unprecedented global burden on public health, socioeconomic systems, and human life.^1^ Although most people affected by COVID-19 have recovered, many have also experienced the profound loss of loved ones.^2^ As of July 2024, there had been more than 700 million confirmed cases and 7 million pandemic-related deaths worldwide.^3^ Despite transitioning from a pandemic to an endemic phase,^4^ COVID-19 continues to pose significant challenges to many countries.^5^ Several spatial and spatiotemporal models, both in the statistical and machine learning (ML) domains, have been used to study the local-level drivers contributing to the dynamics of the disease’s outcomes worldwide,^6-8^ and a systematic review of the current knowledge can help researchers refine approaches adopted to influence decision-making and public health guidelines.^9^^,^^10^

Spatial and spatiotemporal statistical models played a crucial role in understanding the dynamics of COVID-19 throughout the pandemic. For instance, Sun et al.^11^ used spatial lag, spatial error, and spatial autoregressive combined models to investigate how spatial structure influenced geographic disparities in COVID-19 period prevalence.^11^ Similarly, Paul et al.^12^ applied a Bayesian hierarchical spatiotemporal model, using the Markov chain Monte Carlo algorithm, to estimate county-level COVID-19 prevalence in the United States.^12^ Liu et al.^13^ used a geographically weighted regression model to explore how factors such as population movement, weather, air quality, and socioeconomic conditions were linked to COVID-19 incidence at the prefecture level in mainland China.^13^ Chen et al.^14^ used a geographically and temporally weighted regression model to analyze the spatiotemporal relationship between COVID-19 spread and population mobility.

In addition to traditional spatial and spatiotemporal statistical models, ML has emerged as a powerful tool for investigating the dynamics of infectious diseases.^15^ Machine learning refers to a set of computational methods that enable algorithms to learn patterns from data.^16^ Unlike traditional statistical approaches, which draw inferences about a population based on a sample, ML focuses on uncovering complex, often nonlinear patterns in data that can be generalized.^17^ Although ML models present particular challenges, such as limited interpretability and weaker integration with domain knowledge frameworks,^18^^,^^19^ they offer enhanced flexibility and the capacity to handle high-dimensional, heterogeneous data, making them particularly valuable in public health applications.^20^ Machine learning techniques can be broadly categorized into supervised learning (whereby models are trained on labeled data), unsupervised learning (which involves identifying patterns or groupings in data without predefined labels), and semi-supervised learning (which develops models using a combination of both labeled and unlabeled data).^16^ These approaches have evolved to account for spatial structure in data. Machine learning models can be applied to both nonspatial and spatial data. Traditional ML approaches typically do not account for spatial dependencies unless explicitly created to do so. On the other hand, spatial ML methods are adapted or designed to incorporate spatial structures, allowing them to capture location-based patterns and dependencies critical for spatial epidemiology.^21^

Traditional ML models, such as random forest and neural networks, have been applied to COVID-19 data, but they often fall short of capturing the intricate spatial and temporal dependencies inherent in disease spread,^22^^,^^23^ To address these limitations, researchers have increasingly explored spatial and spatiotemporal ML models, including geographically random forest (GRF), spatiotemporal graph neural networks (GNNs), and self-organizing maps, to uncover the complex dynamics of COVID-19.^24-33^ For instance, Grekousis et al.^25^ used the GRF to explore the spatial variations in the nonlinear relationships between COVID-19 death rate and multiple societal and health factors for the first year of the pandemic in the United States.^25^ Croft et al.^24^ proposed a novel GNN architecture for forecasting reported COVID-19 infections in the Netherlands.^24^ This model captures the spatial interaction between municipalities using Graph Attention Networks, version 2, and the spread of COVID-19 within municipalities over time using gated recurrent unit networks.^24^ Additionally, Melin et al.^27^ used self-organizing maps to analyze the spatial evolution of the COVID-19 pandemic worldwide, spatially grouping countries with similar COVID-19 cases severity. Numerous studies have reviewed the application of spatial and spatiotemporal models for studying COVID-19 dynamics. For example, Franch-Pardo et al.^8^ reviewed how geographic and geospatial analyses have been used to understand COVID-19 locations and distribution patterns.^8^ Odhiambo et al.^6^ conducted a systematic review to identify the spatial and spatiotemporal models applied to COVID-19 in Africa, examining risk factors associated with COVID-19 risk in the region.^6^ Nazia et al.^7^ systematically reviewed the spatial and spatiotemporal models used to identify spatial variations of COVID-19 incidence and associated socioeconomic, demographic, and climatic risk factors for such spatial variations.^7^ Fatima et al.^34^ conducted a scoping review of models and findings related to COVID-19, highlighting links to sociodemographic and environmental characteristics.^34^ Aboalyem and Ismail^35^ reviewed different spatial models used for regional COVID-19 data systems, comparing their applications.^35^ And Ahasan et al.^36^ systematically reviewed the use of geographic information system (GIS) technology and other geospatial tools in COVID-19 studies.^36^

Despite these efforts, none of the existing reviews we found thoroughly examined the application of spatial and spatiotemporal ML models for analyzing COVID-19 dynamics. We address this gap here by reviewing works that leverage these models to understand the dynamics of COVID-19 outcomes, and we explore the local-level drivers of these dynamics in the demographic, socioeconomic, environmental, epidemiological, health care, housing conditions, behavioral, and vaccination domains. Although statistical models remain central to spatial epidemiology, our focus on ML approaches is intended to complement this broader body of work by highlighting emerging trends, methodological choices, and reporting practices specific to ML. This review not only identifies key knowledge gaps but also aims to inform and strengthen future research designs, which are crucial for effective public health preparedness in the face of future health threats.

## Methods

### Search strategy

This review adhered to the Preferred Reporting Items for Systematic Reviews and Meta-Analyses (PRISMA) guidelines.^37^^,^^38^ A comprehensive search was conducted across multiple databases, including Scopus, Web of Science, PubMed, the World Health Organization COVID-19 database, and Emcare (via Ovid). Two iterative search strategies used a combination of keywords (Table 1). The initial search was performed in October 2023; we conducted an updated search in May 2024 to capture emerging evidence. Due to technical limitations, Scopus was excluded from the updated search, and the World Health Organization COVID-19 database was no longer accessible. The search was restricted to journal articles published between December 2019 and April 2024. All identified records were imported into Rayyan^39^ for screening and management. Additional studies were retrieved through manual searches of reference lists.

**Table 1:** Search strategy for the search across the 5 databases.

(Coronavirus^*^ OR COVID^*^ OR Alphacoronavirus^*^ OR “Alphacoronavirus 1” OR Betacoronavirus^*^ OR “Betacoronavirus 1” OR Deltacoronavirus^*^ OR Gammacoronavirus^*^ OR “COVID-19” OR “Sars-CoV-2” OR “Covid 19” OR “Cov-19” OR “Coronavirus Infection” OR “Infectious Bronchitis Virus” OR “Coronavirus Infection Disease 2019” OR “2019 Novel Coronavirus Infection” OR “2019-nCoV Infection” OR “2019 nCoV Infection” OR “2019-nCoV Infections” OR “Novel Coronavirus Pneumonia” OR “2019 Novel Coronavirus” OR “Coronavirus Disease 2019” OR nCoV^*^ OR “Bat Coronavirus” OR “COVID-19 Pandemic” OR “COVID-19 Pandemic” OR “Sars-CoV-2 Pandemic” OR “Coronavirus Pandemic” OR “nCoV Pandemic” OR “Coronavirinae”) AND (Mapping OR Spread^*^ OR Variation OR Distribution OR Cluster^*^ OR Pattern^*^ OR Hotspot^*^) AND (Spatia^*^ OR Spatio^*^ OR Space OR “Space–Time” OR “Space Time” OR Forecast^*^ OR Predict^*^ OR Geos^*^ OR Geographic^*^ OR “Geographically Weighted” OR Loca^*^ OR “Locally Weighted”) AND (“Machine Learning” OR “Deep Learning” OR “Artificial Intelligence” OR “Neural Network” OR “Multilayer Perceptron” OR “Random Forest” OR “Gradient Boosting” OR “Boosted Regression” OR “Naive Bayes” OR “Decision Trees” OR “K-Nearest Neighbor” OR “Support Vector Machine” OR “Space–Time Random Forest” OR ML OR DL OR AI OR ANN OR CNN OR RNN OR MLP OR RF OR GNN OR KNN OR SVM OR XGBoost OR LightGBM OR AdaBoost OR LSTM)

### Selection criteria

**Figure 1:**
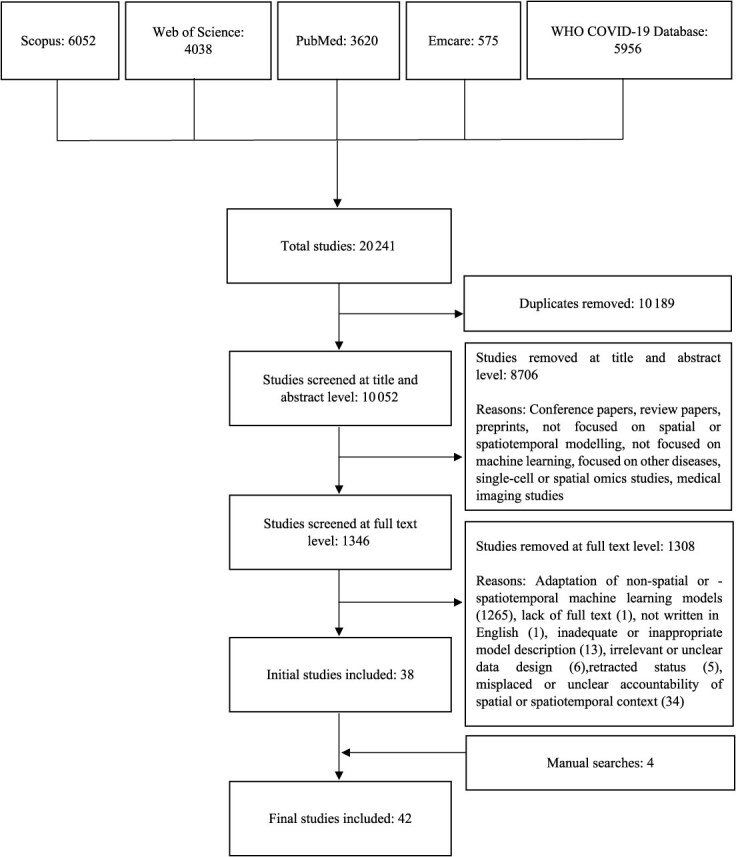
PRISMA diagram for the review selection process. Abbreviation: WHO, World Health Organization.

Studies were included if they were peer-reviewed publications in which authors reported using spatial or spatiotemporal ML models to investigate the dynamics of COVID-19 outcomes. A spatial ML model was defined as one that explicitly or inherently considers spatial context, whereas a spatiotemporal ML model was defined as one that simultaneously considers both spatial and temporal contexts.^40^ Only English-language studies were included. We did not impose geographic restrictions in the article searches. A detailed overview of inclusion and exclusion criteria is presented in Figure 1.

### Quality appraisal tool

The methodological quality of included studies was evaluated using an adapted and validated quality appraisal tool^6^ (Table S1). This instrument evaluated studies based on research aims, data, models, results, and discussions, with a maximum score of 16 points. Studies were categorized into 4 quality levels: very high (score >13), high (11-13), medium (8-10), and low (score <8) (Table S2). Two independent reviewers conducted the assessment, and any disagreements were resolved through consensus.

### Data extraction

Data were extracted from included studies according to the Critical Appraisal and Data Extraction for Systematic Reviews of Prediction Modeling Studies (CHARMS) checklist.^41^ Information collected included study characteristics (title, first author, publication year, study area), research objectives, COVID-19 outcomes, local-level drivers and their characteristics, spatial and spatiotemporal models, models’ characteristics, analytic focus, evaluation metrics, and software and programming languages (Tables 2 and 3).

**Table 2:** Summary of local-level drivers used in the included studies.

**First author, year**	**Study area**	**COVID-19 outcome**	**Local-level drivers**	**Characteristics of local-level drivers** ^**a**^
Murphy (2021)	Spain	Incidence	Population of province, origin-destination network of individual mobility	S
Anno (2022)	Global	Confirmed cases	Airway, railway, and road connections	S
Yu (2021)	United States: nationwide and California	Infected cases	–	–
Li (2023)	Hubei Province, China	Confirmed cases	Population migration index	S
Pu (2023)	Brazil, China, and Austria	Infected cases	Public health intervention (quarantine) metric	S
Sciannameo (2022)	Reggio Emilia, Italy	Cases, hospital admissions	Inhalable particulate matter, PM_2.5_, nitrogen dioxide air concentration, temperature, relative humidity, wind speed, solar radiation, crowding index	S, C
Paul (2020)	United States and Italy	Infection cases	–	–
Muñoz-Organero (2023)	Madrid, Spain	Incidence value	Bike-sharing service (no. of bike stations)	S
Lucas (2023)	United States	Incidence	Average minimum temperature, average maximum temperature, population density, the proportion of Black residents, proportion of Hispanic residents, the proportion of Indigenous residents, proportion of residents older than 65 years, rural land as a proportion of the county area, median household income, stay-put index, movement index	S
Duarte (2023)	Mainland Portugal	Incidence rate	–	–
La Gatta (2020)	Italy	Region: infected, recovered, deceased cases Province: infected cases	Population size, population density, percentage of healthy people, percentage of people at least 1 chronic disease, number of swab tests, fraction of people who stay at home, fraction of incoming people	S
Melin (2020)	Global, Mexico	Confirmed cases, recovered cases, death cases	–	–
Geng (2022)	Massachusetts (United States)	Confirmed cases per 1000 population	–	–
Olsen (2022)	London, United Kingdom	Confirmed cases	Precipitation, temperature, nitrogen oxide concentration, population, population density, percentage of population aged 65 years or older, percentage of population from Black, Asian, and minority ethnic backgrounds, individuals per dwelling, stringency index	S, C
Fritz (2022)	Germany	Cases (infections)	Population, population density, sex/age group, distance network, percentage of people staying put	S
Paul (2021)	United States	Incidence rate	Population density, housing density, female fraction, median age, average daily temperature, temperature deviation, average daily relative humidity	S
Jia (2022)	Los Angeles, CA (United States)	Infection number (cases)	Population, social vulnerability index, visit patterns, social distance metrics	S, C
Oliveira (2022)	Brazil	Cases, deaths	Population, demographic density, capacity for caring sick people	S
Yudistira (2021)	Global: 55 countries for training and 4 countries for testing	Confirmed cases, recovered cases, death cases	Population, population density, median age, age ≥65 years, age ≥70 years, gross domestic product per capita, female smokers, male smokers, extreme poverty, cardiovascular death rate, diabetes prevalence, hospital beds per thousand, life expectancy, human development index, average cloud modification factor, ozone column, stringency index	S, C
Cardoso (2022)	Mainland Portugal	Incidence rate	–	–
Huang (2022)	Europe: Germany, Italy, and Spain	Confirmed cases	–	–
Skianis (2023)	France	Positive cases, hospitalized patients	Population of department, vaccination rate, population movement	S
Melin (2021)	Global	Confirmed cases, recovered cases, death cases	–	–

**Table 2:** Continued

**First author, year**	**Study area**	**COVID-19 outcome**	**Local-level drivers**	**Characteristics of local-level drivers** ^**a**^
Banerjee (2022)	United States: Nationwide and Michigan	Infected cases, death cases	–	–
Wang (2023)	Scotland, United Kingdom	Confirmed positive cases per 100 000 population	Proportion of female individuals, proportion of people aged >60 years, mental health prescriptions (ratio), drug-related hospitalizations (ratio), proportion of White people, alcohol-related hospitalizations (ratio), health and social workers (ratio), income (British pounds), education (ratio), language skill (ratio), religion (ratio), population density, urbanity, crowded household (ratio), energy consumption median, broadband accessibility (ratio), per-capita area of public green space, crime (ratio per 10 000 people), annual average ambient concentrations of nitrogen monoxide, annual average ambient concentrations of fine particulate matter, annual average ambient concentrations of sulfur monoxide, standardized mortality ratio	S
Galvan (2021)	Brazil	Cases and deaths per 100 000 inhabitants	–	–
Wang (2021)	Ohio, United States	Risk/prevalence	Population, population density, median age, age distribution, sex distribution, individuals per household	S
Niraula (2022)	Castilla-Leon, Spain	Infected cases	Total population of health zones, number of people demanding employment, number of people registered as unemployed, number of urban commercial offices in the urban areas, number of industrial units in the urban areas, number of offices units in the urban areas, daily human mobility	S
Wang (2023)	China	Confirmed cases	Population density, migration index	S
Muñoz-Organero (2022)	Madrid, Spain	Incidence	–	–
Ramchandani (2020)	United States	Infected cases	Age/sex, race, ethnicity, household/family type, school enrollment, language spoken at home, poverty status, income, employment status and occupation, county population, county population density, socioeconomic vulnerability index, household composition and disability, minority status and language, housing type and transportation, epidemiological vulnerability, health care system factors, COVID-19 community vulnerability index, colleges/universities/professional schools, living facilities for elderly, hospitals, percentage of people working full- or part-time, concentration of population activities in a county, social distancing metrics, Venables distance, cross-county mobility, total visit to points of interests, percentage of people staying home	S, C

**Table 2:** Continued

**First author, year**	**Study area**	**COVID-19 outcome**	**Local-level drivers**	**Characteristics of local-level drivers** ^**a**^
Luo (2021)	United States	Mortality rate	Airborne concentrations of PM_2.5_, benzene, formaldehyde, acetaldehyde, carbon tetrachloride, air temperature, precipitation, sunlight exposure, ultraviolet radiation exposure, drought, flood, disability, asthma, obesity, overweight, cancer, health insurance, householder with a mortgage, poverty, service occupation, unemployment, number of hospitals, number of hospital beds per 10 000 population, people living in group quarter, householder with no internet access, median household income, mean household retirement income, mean household cash public assistance income, mean household supplemental security income, and percentages of the following: male individuals, median age, percentage of people aged <18 years, of people ≥65 years, of White race, Black or African American race, American Indian and Alaska native, Asian race, Native Hawaiian and other Pacific Islander, Hispanic or Latino	S
Grekousis (2022)	United States	Deaths per 100 000 population	Population density, % by age: 20-39 years, 40-59 years, 60-79 years, ≥80 years % Population by race: Black or African American alone, Asian alone Percentages of the following: civilian noninstitutionalized population with a disability, average household size, occupied housing units with no vehicles available, housing problem, population aged ≥25 years, bachelor’s degree, work construction and trade sector, work services sector, work social sector, median income, unemployment, no insurance, poverty, heart disease mortality, % with asthma, obesity, sleep <7 hours, no leisure-time physical activity, smokers	S
Wang	Germany	Prevalence	–	–
Gao	United States	Infected cases	Population size, population density	S
Zhang	Denver metropolitan area, Colorado, United States	Infected cases	Population, percentage of population in poor health, number of individuals in poverty, unemployment in civil labor force, weekday traffic flows, weekend traffic flows	S
Sun (2022)	Global	Cases	Vaccination rate, flight count	S
Ravenda (2024)	Italy	Deaths	–	–
Zhou (2020)	United States	Prevalence	Population size, mobility reduction of individuals during the epidemic	S
Muñoz-Organero (2022)	Madrid, Spain	Incidence	Traffic-driven mobility in adjacent districts	-
Croft (2023)	The Netherlands	Incidence	Population density, virus load in sewage water, stringency index	S, C
Zhao (2023)	New York, NY, and San Francisco, CA, United States	Cases per 100 000 people	Median household income, population density, residents’ average age, monthly averaged traffic density	S

Abbreviations: C, composite indicator; PM_2.5_, fine particulate matter; S, standalone factor. Dash lines, No local-level driver within the predefined contextual domains.

**Table 3:** Summary of machine learning models used in the included studies.

**First author, year**	**Spatial or/and spatiotemporal ML method (main)**	**Spatial or/and spatiotemporal ML method (baseline)**	**Characteristics of method**				**Analytic focus**	**Evaluation metric**	**Software or programming language**
Murphy (2021)	GAT + RNN	CovidGNN	GAT + RNN	G	ST	H	P	PCC	Python
			CovidGNN	G	ST	H			
Anno (2022)	3-layer spatiotemporal GCN	–	3-Layer Spatiotemporal GCN	G	ST	NH	P	MSE, RMSE, MAE, RMSPE, MAPE	–
Yu (2021)	IT-GCN	STGCN (Yu)	IT-GCN	G	ST	H	P	MAE, RMSE, MAPE	Python
			STGCN (Yu)	G	ST	H			
Li (2023)	LsOA + GCN	LsOA + GCN, T-GCN, GAT, GCN	LsOA + GCN	G	ST	H	P	RMSE, MAE, MAPE, Acc, *R*^2^, Var	Python 3.6.12: TensorFlow 1.14.0
			T-GCN	G	ST	H			
			GAT	G	S	NH			
			GCN	G	S	NH			
Pu (2023)	DASTGN	GCN, STGCN (Yu), Graph WaveNet, ColaGNN, STSGCN, Ada-STNet	DASTGN	G	ST	H	P	MSE, RMSE, MAE	Python: PyTorch 1.10.1
			GCN	G	S	NH			
			STGCN (Yu)	G	ST	H			
			Graph WaveNet	G	ST	H			
			ColaGNN	G	ST	H			
			STSGCN	G	ST	H			
			USTGCN	G	ST	NH			
			Ada-STNet	G	ST	H			
Sciannameo (2022)	ConvLSTM (Shi)	–	ConvLSTM (Shi)	G	ST	H	P	MOV, MAE, RMSE	R: Keras R Interface using TensorFlow
Paul (2020)	ConvLSTM (Paul ‘20)	–	ConvLSTM (Paul ‘20)	G	ST	H	P	MAPE, KL Divergence	–
Muñoz-Organero (2023)	Space-distributed LSTM	Deep hybrid LSTM, space-distributed Traffic-Enhanced LSTM	Space-distributed LSTM	G	ST	NH	P	RMSE, MAPE	–
			Deep hybrid LSTM-CNN	G	ST	H			
			Space-distributed traffic-enhanced LSTM	G	ST	NH			
Lucas (2023)	COVID-LSTM	–	COVID-LSTM	G	ST	NH	P	MAE, MAPE	Python: TensorFlow 2.5.0
Duarte (2023)	Symbolic regression with stochastic sequential simulation	–	Symbolic regression with stochastic sequential simulation	G	ST	NH	A	*R* ^2^	–
La Gatta (2020)	GCN + LSTM + SIR, GCN + LSTM + SIRD	–	GCN + LSTM + SIR	G	ST	H	P	RPE	Python: PyTorch and PyTorch Geometric Library
			GCN + LSTM + SIRD	G	ST	H			
Melin (2020)	SOM	–	SOM	G	S	NH	C	–	–

**Table 3:** Continued

**First author, year**	**Spatial or/and spatiotemporal ML method (main)**	**Spatial or/and spatiotemporal ML method (baseline)**	**Characteristics of method**				**Analytic focus**	**Evaluation metric**	**Software or programming language**
Geng (2022)	SGWT + GAT	–	SGWT + GAT	G	ST	H	A	Acc	Python: Scikit Learn Library
Olsen (2022)	LSTM-ANN	–	LSTM-ANN	G	ST	H	P	*R* ^2^, RMSE, NRMSE	–
Fritz (2022)	Zero-inflated Poisson distributional regression + GNN, negative binomial distributional regression + GNN	GNN	Zero-inflated Poisson distributional regression + GNN	G	ST	H	P	RMSE	–
			Negative binomial distributional regression + GNN	G	ST	H			
			GNN	G	S	NH			
Paul (2021)	ConvLSTM (Paul ‘21)	ConvLSTM (Paul ‘20)	ConvLSTM (Paul ‘21)	G	ST	H	D, P	MAPE, MSE, KL Divergence	Python: Scipy
			ConvLSTM (Paul ‘20)	G	ST	H			
Jia (2022)	FGC-COVID	LSTNET, CNNRNN_Res, DCRNN, STGCN (Yu), GoogleGNN, ColaGNN, MPNN + LSTM	FGC-COVID	G	ST	H	P	MAE, RMSE, WMAPE	Python: PyTorch and PyTorch-Geometric Library
			LSTNET	G	ST	H			
			CNNRNN_Res	G	ST	H			
			DCRNN	G	ST	H			
			STGCN (Yu)	G	ST	H			
			GoogleGNN	G	ST	H			
			ColaGNN	G	ST	H			
			MPNN + LSTM	G	ST	H			
Oliveira (2022)	STGCN (Oliveira)	–	STGCN (Oliveira)	G	ST	H	P	MAE, sMAPE, NRMSEsd	QGIS, Python: PyTorch and PyTorch Geometric v1.8.1
Yudistira (2021)	ConvLSTM (Yudistira)	CNN	ConvLSTM (Yudistira)	G	ST	H	D, P	RMSE	Python
			CNN	G	S	NH			
Cardoso (2022)	STConvS2S	ConvLSTM (Shi)	STConvS2S	G	ST	H	P	RMSE, sMAPE	Python
			ConvLSTM (Shi)	G	ST	H			
Huang (2022)	COVID-19Net	CNN, CNN-GRU	COVID-19Net	G	ST	H	P	MAE, RMSE, MAPE	Python 3.7: TensorFlow
			CNN	G	S	NH			
			CNN-GRU	G	ST	H			
Skianis (2023)	Multi-scale MPNN	MPNN, MPNN + LSTM	Multi-scale MPNN	G	ST	H	P	MAE	–
			MPNN	G	S	NH			
			MPNN + LSTM	G	ST	H			
Melin (2021)	SOM, SOM + fuzzy fractal	–	SOM	G	S	NH	C, P	Acc	Fractalyse 2.4.1, Matlab R2018b Language
			SOM + Fuzzy Fractal	G	ST	H			

**Table 3:** Continued

**First author, year**	**Spatial or/and spatiotemporal ML method (main)**	**Spatial or/and spatiotemporal ML method (baseline)**	**Characteristics of method**				**Analytic focus**	**Evaluation metric**	**Software or programming language**
Banerjee (2022)	STSGT Network	STGCN (Yu), ASTGCN, Graph WaveNet, STTN	STSGT Network	G	ST	H	P	MAE, RMSE, RMSLE	Python: PyTorch 1.8.0
			STGCN (Yu)	G	ST	H			
			ASTGCN	G	ST	H			
			Graph WaveNet	G	ST	H			
			STTN	G	ST	H			
Wang (2023)	GRF	–	GRF	L	S	NH	D	*R* ^2^, RMSE, MAE	R: SpatialML Library
Galvan (2021)	SOM	–	SOM	G	S	NH	C	MQE	Matlab
Wang (2021)	MK-DNN	–	MK-DNN	G	S	H	P	MSE, Acc	Python: Keras
Niraula (2022)	LSTM-INLA	–	LSTM-INLA	G	ST	H	P	RMSE, DIC, WAIC, CPO	Python, R
Wang (2023)	LSTM-CA	–	LSTM-CA	G	ST	H	P	Statistical Acc, spatial Acc	ArcGIS 10.5; Python 3.4: GDAL 3.3.0, Numpy 1.21, Matplotlib 3.3.2, Scikit-Learn 0.23.2
			Spatial CA	G	S	NH			
Muñoz-Organero (2022)	Spatiotemporal CNN-RNN	CNN	Spatiotemporal CNN-RNN	G	ST	H	P	RMSE, EV	Python
			CNN	G	S	NH			
Ramchandani (2020)	DeepCOVIDNet	–	DeepCOVIDNet	G	ST	H	D, P	Acc	Python
Luo (2021)	GW-RF	–	GW-RF	L	S	NH	D	MSE, *R*^2^	R 3.5.3
Grekousis (2022)	GRF	–	GRF	L	S	NH	D	*R* ^2^, RMSE, MAE	R: randomForest and SpatialML
Wang (2021)	CA + modified SUIR	–	CA + modified SUIR	G	ST	H	P	MSE, *R*^2^	Python
Gao (2021)	STAN	ColaGNN, CovidGNN	STAN	G	ST	H	P	MSE, MAE, CCC	Python
			ColaGNN	G	ST	H			
			CovidGNN	G	ST	H			
Zhang (2021)	DCRNN + GCN + LSTM + SIR	–	DCRNN + GCN + LSTM + SIR	G	ST	H	P	MAE, MAPE	Python: Numpy, Scikit Learn, Keras 2.1, Network 2.4
Sun (2022)	GAT + SIRVC, GAT + MLP + SIRVC	ColaGNN, CovidGNN, STAN	GAT + SIRVC	G	ST	H	P	MSE, RMSE	Python
			GAT + MLP + SIRVC	G	ST	H			
			ColaGNN	G	ST	H			
			CovidGNN	G	ST	H			
Ravenda (2024)	PNN	–	PNN	G	ST	H	P	MAE, MSE	–
Zhou (2020)	CA-eSAIR	–	CA-eSAIR	G	ST	H	P	WPE, WAPE	R

**Table 3:** Continued

**First author, year**	**Spatial or/and spatiotemporal ML method (main)**	**Spatial or/and spatiotemporal ML method (baseline)**	**Characteristics of method**				**Analytic focus**	**Evaluation metric**	**Software or programming language**
Muñoz-Organero (2022)	Space-distributed traffic-enhanced LSTM	–	Space-distributed traffic-enhanced LSTM	G	ST	NH	P	MSE, MAPE	Python: Keras Library
Croft (2023)	GATv2 + GRU	–	GATv2 + GRU	G	ST	H	P	RMSE, *R*^2^	Python: PyTorch and PyTorch Geometric Library
			GATv2	G	S	NH			
Zhao (2023)	CHGCN	TRI, C-MPGCN	CHGCN	G	S	NH	P	Acc, MRR, HR@k	–

Abbreviations: A, anomaly; Acc, accuracy; CCC, concordance correlation coefficient; ColaGNN, cross-location attention-based graph neural network; CNN, convolutional neural network; CPO, conditional predictive ordinate; D, Descriptive; G, global; GATv2, Graph Attention Networks, version 2; GRU, gated recurrent unit [network]; H, hybrid; HR@k, hit ratio at K; KL, Kullback–Leibler; L, local; LSTM, long short-term memory; MAE, mean absolute error; MAPE, mean absolute percentage error; MK-DNN, multikernel density estimation and deep neural network; ML, machine learning; MOV, mean observed value; MPNN, message passing neural network; MQE, mean quantization error; MRR, mean reciprocal rank; MSE, mean square error; NH, not hybrid; P, predictive; PCC, Pearson correlation coefficient; RMSE, root mean square error; RMSLE, root mean squared logarithmic error; RMSPE, root mean squared percentage error; RPE, relative prediction error; S, spatial; sMAPE, symmetric mean absolute percentage error; ST, spatiotemporal; STGCN, spatiotemporal graph convolutional neural network; WAIC, Watanabe-Akaike information criterion; WAPE, weighted absolute prediction error; WMAPE, weighted mean absolute percentage error; WPE, weighted prediction error; CHGCN, contrastive learning-based hierarchical graph convolutional neural network; TRI, triplet network; C-MPGCN, contrastive learning-based multi-relational graph convolutional neural network; Ca-eSAIR, Temporal extended susceptible-antibody-infectious-removed model with Cellular Automata; PNN, probabilistic neural network; CovidGNN, spatio-temporal graph neural network; MLP, multilayer perceptron; SIRVC, susceptible-infected-recovered with vaccinations and inter-community interaction; GAT, graph attention network; STAN, spatio-temporal attention network; SIR, susceptible-infected-recovered; DCRNN, diffusion convolutional recurrent neural network; GCN, graph convolutional network; RNN, recurrent neural network; SUIR, sensitive-undiagnosed-infected-removed; DeepCOVIDNet, deep spatio-temporal neural network for COVID-19 forecasting; CNN-RNN, convolutional-recurrent neural network hybrid; LSTM-CA, long short-term memory with cellular automata; LSTM-INLA, long short-term memory with integrated nested laplace approximation; GRF, geographical random forest; GW-RF, geographically weighted random forest; ASTGCN, attention-based spatial-temporal graph convolution network; STSGT, spatial-temporal synchronous graph transformer network; STTN, spatio-temporal transformer network; CNN-GRU, convolutional neural network with gated recurrent unit; COVID-19Net, novel spatiotemporal feature extraction parallel deep neural network; ConvLSTM, convolutional long-short term memory; GoogleGNN, google graph neural network; FGC-COVID, fine-grained population mobility data-based community-level COVID-19 prediction model; LSTNET, long- and short-term time-series network; CNNRNN_Res, convolutional and recurrent residual hybrid network; COVID-LSTM, long short-term memory network for COVID-19 forecasting; Ada-STNet, adaptive spatio-temporal graph neural network; USTGCN, unified spatio-temporal graph convolution network; STSGCN, spatio-temporal synchronous graph convolutional network; SGWT, spectral graph wavelet transform; DASTGN, dynamic adaptive spatio-temporal graph network; T-GCN, temporal graph convolutional network; LsOA, lioness optimal algorithm; IT-GCN, interaction-temporal graph convolutional network; EV/Var, explained variance score; SIRD, susceptible-infected-recovered-deceased; ANN, Artificial neural network; GNN, Graph neural network; STConvS2S, Spatio-temporal convolutional sequence-to-sequence neural network; NRMSEsd, normalized root mean square error by standard deviation; NRMSE, normalized root mean square error.

## Results

### Review summary

A comprehensive search yielded 20 241 initial records, which were reduced to 10 052 distinct articles after removing duplicates (Figure 1). Title and abstract screening excluded 8706 studies, leaving 1346 for full-text assessment. Of these, 1308 were deemed ineligible, resulting in 38 included articles. Manual searches identified 4 additional studies, bringing the final count to 42 (Figure 1, Tables 2 and 3). The mean quality score of the included studies was 14.62 out of a possible 16, indicating high overall quality (Table S3).

### Temporal and geographic scope of the included studies

**Figure 2:**
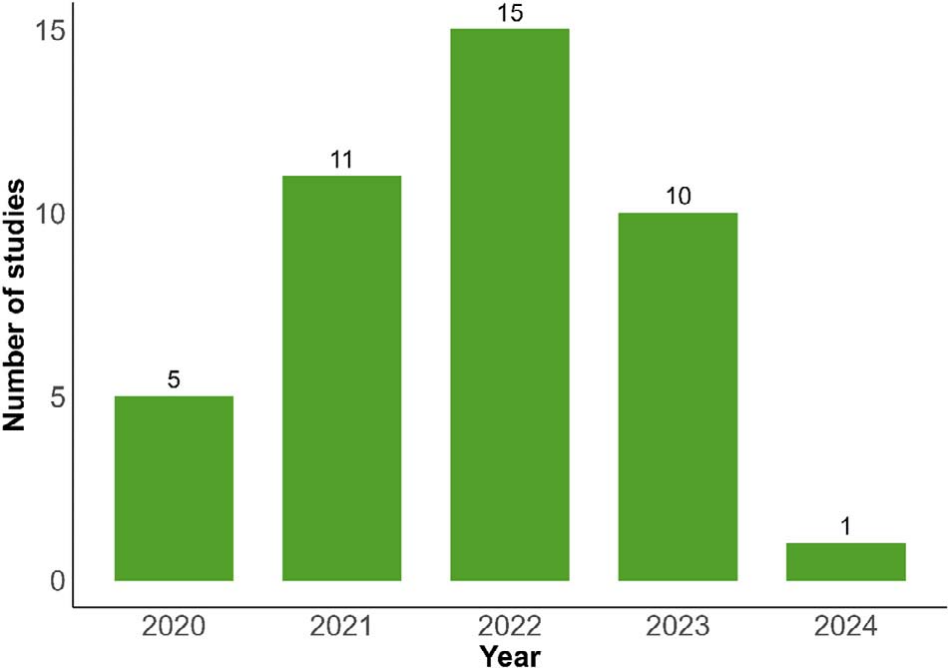
Number of studies by year.

**Figure 3:**
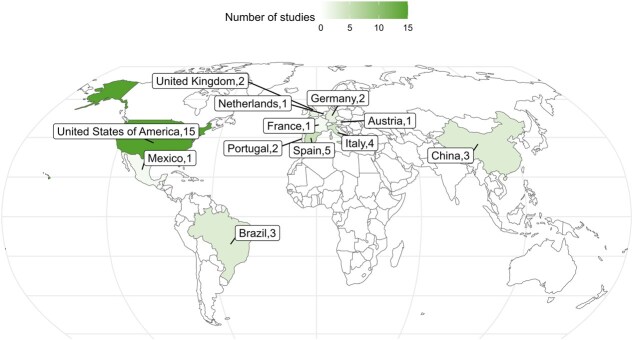
Number of studies by geographic location (excluding continental and global studies.

The distribution of studies in this review varied across different scopes. The publication year distribution of included studies spanned from 2020 to 2024, with a concentration in 2022 (Figure 2). The United States dominated geographically, followed by Spain, Italy, Brazil, and China (Figure 3).

### COVID-19 outcomes

**Figure 4:**
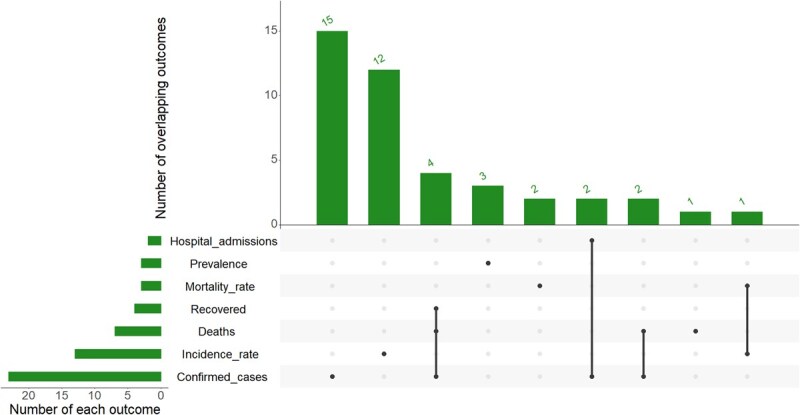
Distribution and overlap of COVID-19 outcome variables used across studies. The left bar plot shows the total number of studies reporting each outcome; the matrix and the vertical bars (top) illustrate the number of studies that reported combinations of outcomes.

COVID-19 outcomes varied across studies (Figure 4). The most common outcome was confirmed cases, used in 23 studies.^27^^,^^28^^,^^32^^,^^42-61^ Others were incidence rate, (*n* = 13 studies)^24^^,^^26^^,^^31^^,^^33^^,^^50^^,^^51^^,^^58^^,^^59^^,^^62–66^; death (*n* = 7 studies)^27^^,^^29^^,^^32^^,^^46^^,^^47^^,^^50^^,^^53^; recovery rates (*n* = 4 studies)^27^^,^^32^^,^^46^^,^^48^; mortality rates (*n* = 3 studies)^25^^,^^30^^,^^64^; risk or prevalence (*n* = 3 studies)^67–69^; and hospital admissions (*n* = 2 studies).^56^^,^^59^ Only a few studies analyzed multiple overlapping outcomes (eg, death, recovery, confirmed cases).

### Local-level drivers

The types and frequency of local-level drivers exhibited substantial heterogeneity (Table 2). To facilitate analysis, drivers were categorized into 10 predefined contexts: demographic, socioeconomic, environmental, epidemiologic, health care, housing, behavioral, vaccination, governmental policy, and mobility. Local-level drivers spanning multiple contexts were classified as composite indicators. Of the 42 included studies, 29 incorporated at least 1 local-level driver from the defined contexts, and 13 did not. Only 6 studies used composite indicators.

Demographic factors commonly reported included population density^24–26^^,^^32^^,^^48–54^^,^^68^^,^^70–72^; population size^32^^,^^48–51^^,^^53–58^^,^^68^^,^^69^^,^^73^; race and ethnic variables (eg, Black, Hispanic, Asian, White, Indigenous)^25^^,^^30^^,^^49^^,^^56^^,^^70^^,^^71^; elderly population^25^^,^^30^^,^^32^^,^^49^^,^^70^^,^^71^; median/average age^26^^,^^30^^,^^32^^,^^65^^,^^69^; sex/age distribution/group^48^^,^^51^^,^^65^; and female proportion.^26^^,^^57^ Environmental variables frequently considered were temperature^26^^,^^30^^,^^49^^,^^59^^,^^71^; fine and inhalable particulate matter^30^^,^^59^^,^^71^; nitrogen oxide concentration^49^^,^^59^^,^^70^; relative humidity^30^^,^^49^; precipitation^30^^,^^49^; and solar radiation.^30^^,^^59^

Socioeconomic factors, such as income,^25^^,^^30^^,^^53^^,^^70–72^ unemployment- and occupation-related variables,^25^^,^^30^^,^^53^^,^^57^^,^^58^ and poverty,^25^^,^^30^^,^^32^^,^^53^^,^^58^ were commonly reported, followed by education- and literacy-related variables,^25^^,^^53^^,^^70^ rurality- and urbanity-related variables,^57^^,^^70^^,^^71^ and language-related variables.^49^^,^^53^ Epidemiologic factors, including asthma,^25^^,^^30^ obesity,^25^^,^^30^ and cardiovascular deaths,^25^^,^^32^ were also considered. Health care factors, such as the number of available hospital beds,^30^^,^^32^^,^^51^ disability,^25^^,^^30^ number of hospitals,^30^^,^^53^ and health insurance,^25^^,^^30^ were prevalent.

Household size emerged as the most prevalent housing condition factor^25^^,^^49^^,^^68^; tobacco smoking was the most prevalent behavioral factor^25^^,^^32^; vaccination rate among the vaccination factors^56^^,^^60^; stay-put index among the governmental policy data^48^^,^^50^^,^^53^^,^^71^; migration/movement index among the mobility data^43^^,^^52^^,^^56^^,^^71^; a stringency index^24^^,^^32^^,^^49^; and a social vulnerability index^53^^,^^55^ among the composite indicators. The list of all the local-level drivers extracted from the reviewed studies is provided in Table 4.

**Table 4:** Summary of the local-level drivers used in studying COVID-19 spatial and spatiotemporal dynamics.

**Context**	**Standalone factors and composite indicator**	**No. of studies**	**References**
	Population/demographic density	15	^24–26^ ^,^ ^32^ ^,^ ^48–54^ ^,^ ^68^ ^,^ ^70–72^
	Population size/number/count	14	^32^ ^,^ ^48^ ^,^ ^53–58^ ^,^ ^68^ ^,^ ^69^
	Race- and ethnicity-related factors	6	^25^ ^,^ ^30^ ^,^ ^49^ ^,^ ^53^ ^,^ ^70^ ^,^ ^71^
	Elderly population	6	^25^ ^,^ ^30^ ^,^ ^32^ ^,^ ^49^ ^,^ ^70^ ^,^ ^71^
	Median/average age	5	^26^ ^,^ ^30^ ^,^ ^32^ ^,^ ^68^ ^,^ ^72^
	Sex/age distribution/group	3	^50^ ^,^ ^53^ ^,^ ^68^
	Female proportion	2	^26^ ^,^ ^70^
	Religion	1	^70^
	Male sex, %	1	^30^
	Working-age adult population	1	^25^
Demographic	Youth population	1	^30^
	Income	6	^25^ ^,^ ^30^ ^,^ ^53^ ^,^ ^70–72^
	Unemployment- and occupation-related factors	5	^25^ ^,^ ^30^^,^ ^53^^,^ ^57^^,^ ^58^
	Poverty	5	^25^ ^,^ ^30^^,^ ^32^^,^ ^53^^,^ ^58^
	Education- and literacy-related factors	3	^25^ ^,^ ^53^ ^,^ ^70^
	Rurality- and urbanity-related factors	3	^57^ ^,^ ^70^ ^,^ ^71^
	Language-related factors	2	^49^ ^,^ ^53^
	Gross domestic product per capita	1	^32^
	Energy consumption median	1	^70^
	Broadband accessibility	1	^70^
Socioeconomic	Crime	1	^70^
	Temperature	5	^26^ ^,^ ^30^ ^,^ ^49^ ^,^ ^59^ ^,^ ^71^
	Fine and inhalable particulate matter	3	^30^ ^,^ ^59^ ^,^ ^70^
	Nitrogen oxide concentrations	3	^49^ ^,^ ^59^ ^,^ ^70^
	Solar radiation/exposure	3	^30^ ^,^ ^59^
	Precipitation	2	^30^ ^,^ ^49^
	Relative humidity	2	^30^ ^,^ ^49^
	Wind speed	1	^59^
	Airborne pollutants, concentration		
	Benzene	1	^30^
	Formaldehyde	1	^30^
	Acetaldehyde	1	^30^
	Carbon tetrachloride	1	^30^
	Annual average ambient concentrations of sulfur monoxide	1	^70^
	Drought	1	^30^
	Flood	1	^30^
	Ozone column	1	^32^
	Ultraviolet radiation/exposure	2	^30^
	Average cloud modification factor	1	^32^
	Virus load in sewage water	1	^24^
Environmental	Per-capita area of public green space	1	^70^
	Asthma	2	^25^ ^,^ ^30^
	Obese	2	^25^ ^,^ ^30^
	Cardiovascular deaths	2	^25^ ^,^ ^32^
	Chronic disease	1	^48^
	Cancer	1	^30^
	Diabetes prevalence	1	^32^
	Overweight	1	^30^
	Percentage of population in poor health	1	^71^
	Drug-related hospitalizations	1	^70^
	Alcohol-related hospitalizations	1	^70^
Epidemiologic	Standardized mortality ratio	1	^70^
	Hospital beds	3	^30^ ^,^ ^32^^,^ ^51^
	Disability	2	^25^ ^,^ ^30^
	No. of hospitals	2	^30^ ^,^ ^53^
	Health insurance	2	^25^ ^,^ ^30^
	Percentage of healthy people	1	^48^
	No. of swab tests	1	^48^
	Mental health prescriptions	1	^70^
	Health and social workers	1	^70^
	Living facilities for elderly	1	^53^ ^,^ ^58^
Health care	Life expectancy	1	^32^ ^,^ ^53^

**Table 4:** Continued

**Context**	**Standalone factors and composite indicator**	**No. of studies**	**References**
	Household size	3	^25^ ^,^ ^49^ ^,^ ^68^
	Housing density	1	^26^
	Crowded household	1	^70^
	Household/family type	1	^53^
	Householder with a mortgage	1	^30^
	People living in group quarters	1	^30^
	Housing units with no vehicles available	1	^25^
	Householder with no internet access	1	^30^
Housing condition	Housing problem	1	^25^
	Smokers	2	^25^ ^,^ ^32^
Behavioral	Sleep <7 hours	1	^25^
	No leisure-time physical activity	1	^25^
Vaccination	Vaccination rate (COVID-19)	2	^56^ ^,^ ^60^
	Stay-put index	4	^48^ ^,^ ^50^ ^,^ ^53^ ^,^ ^71^
Governmental policy	Social distance metrics	2	^53^ ^,^ ^55^
	Quarantine metric	1	^44^
	Migration/movement index	4	^43^ ^,^ ^52^ ^,^ ^56^ ^,^ ^71^
	Visit patterns	2	^53^ ^,^ ^55^
	Traffic flow/density (weekly and monthly)	2	^58^ ^,^ ^72^
	Origin-destination network of individual mobility	1	^73^
	Airway, railway, and road connections	1	^28^
	Bike-sharing service (no. of bike stations)	1	^62^
	Fraction of incoming people	1	^48^
	Distance network	1	^50^
	Venables distance	1	^53^
	Cross-county mobility	1	^53^
	Flight count	1	^60^
Mobility	Mobility reduction of individuals during the epidemic	1	^69^
	Stringency index	3	^24^ ^,^ ^32^^,^ ^49^
	Social vulnerability index	2	^53^ ^,^ ^55^
	Crowding index	1	^59^
	Human development index	1	^32^
	Epidemiological vulnerability index	1	^53^
	COVID-19 community vulnerability index	1	^53^
	Household composition and disability	1	^53^
	Minority status and language	1	^53^
	Housing type and transportation	1	^53^
Composite indicator	Health care system factors	1	^56^

### Spatial and spatiotemporal ML models

A diverse range of spatial and spatiotemporal ML models was used in the included studies. To facilitate analysis, models were categorized based on several dimensions: local or global and hybrid or nonhybrid. In this study, a global model refers to one that uses uniform parameters across the entire region, whereas a local model allows these parameters to vary across sub-regions to capture spatial heterogeneity. A hybrid ML model refers to the combination of an ML algorithm with other modeling techniques, including ML, statistical, and mechanistic models, to leverage their complementary strengths.

No studies exclusively used hybrid spatial and spatiotemporal models at a local level. However, 3 studies incorporated nonhybrid spatial models, specifically the GRF. These studies explored the relationship between COVID-19 outcomes and various drivers at a local level.^25^^,^^30^^,^^70^

On the other hand, several studies used global spatial and spatiotemporal ML models to analyze COVID-19 dynamics, with most falling under the hybrid category (Table 5). The only spatial ML model used in the hybrid category was the multikernel density estimation and deep neural network (1 study).^68^ The commonly used spatiotemporal ML models included spatiotemporal graph convolutional neural networks (*n* = 5 studies)^42^^,^^44^^,^^47^^,^^55^; cross-location attention-based GNN (*n* = 4 studies)^44^^,^^54^^,^^55^^,^^60^; CovidGNN (*n* = 3 studies)^54^^,^^60^^,^^73^; 2 conventional convolutional long short-term memory (ConvLSTM) models (Shi; *n* = 2 studies),^31^^,^^59^ ConvLSTM (Paul ’20; *n* = 2 studies)^26^^,^^45^; graph wavenet (*n* = 2 studies)^44^^,^^47^; message passing neural network with LSTM (*n* = 2 studies)^55^^,^^56^; and spatiotemporal attention network (*n* = 2 studies).^54^^,^^60^ In the nonhybrid category, commonly used spatial ML models included diverse GNN architectures, diverse convolutional neural network architectures, and self-organizing maps. Space-distributed traffic-enhanced LSTM was frequently used as a spatiotemporal ML model.

**Table 5:** Summary of the global spatial and spatiotemporal ML models.

**Category**	**Model name/composition**	**No. of studies**	**References**
**Hybrid**			
Spatial analysis	MK-DNN	1	^68^
Spatiotemporal analysis	STGCN (Yu)	4	^42^ ^,^ ^44^^,^ ^47^^,^ ^55^
	ColaGNN	4	^44^ ^,^ ^54^^,^ ^55^^,^ ^60^
	CovidGNN	3	^54^ ^,^ ^60^ ^,^ ^73^
	ConvLSTM (Shi)	2	^31^ ^,^ ^59^
	ConvLSTM (Paul ‘20)	2	^26^ ^,^ ^45^
	Graph WaveNet	2	^44^ ^,^ ^47^
	MPNN + LSTM	2	^55^ ^,^ ^56^
	STAN	2	^54^ ^,^ ^60^
	IT-GCN	1	^42^
	LsOA + GCN	1	^43^
	T-GCN	1	^43^
	DASTGN	1	^44^
	STSGCN	1	^44^
	Ada-STNet	1	^44^
	GAT + RNN	1	^73^
	Deep hybrid LSTM-CNN	1	^62^
	GCN + LSTM + SIR	1	^48^
	GCN + LSTM + SIRD	1	^48^
	SGWT + GAT	1	^33^
	LSTM-ANN	1	^49^
	Zero-inflated Poisson distributional regression + GNN	1	^50^
	Negative binomial distributional regression + GNN	1	^50^
	FGC-COVID	1	^55^
	LSTNET	1	^55^
	CNNRNN_Res	1	^55^
	DCRNN	1	^55^
	GoogleGNN	1	^55^
	STGCN (Oliveira)	1	^51^
	ConvLSTM (Yudistra)	1	^32^
	ConvLSTM (Paul ‘21)	1	^26^
	STConvS2S	1	^31^
	COVID-19Net	1	^61^
	CNN-GRU	1	^61^
	Multiscale MPNN	1	^56^
	SOM + fuzzy fractal	1	^46^
	STSGT Network	1	^47^
	ASTGCN	1	^47^
	STTN	1	^47^
	LSTM-INLA	1	^57^
	LSTM-CA	1	^52^
	Spatiotemporal CNN-RNN	1	^63^
	DeepCOVIDNet	1	^53^
	CA + modified SUIR	1	^67^
	STAN	1	^57^
	DCRNN + GCN + LSTM + SIR	1	^58^
	GAT + SIRVC	1	^60^
	GAT + MLP + SIRVC	1	^60^
	PNN	1	^29^
	CA-eSAIR	1	^69^
	GATv2 + GRU	1	^24^
**Nonhybrid**			
Spatial analysis	Diverse GNN architectures	6	^24^ ^,^ ^43^ ^,^ ^44^ ^,^ ^50^ ^,^ ^56^ ^,^ ^72^
	Diverse CNN architectures	4	^32^ ^,^ ^53^ ^,^ ^61^ ^,^ ^72^
	SOM	3	^27^ ^,^ ^46^ ^,^ ^64^
	Spatial CA	1	^52^
	Symbolic regression with stochastic sequential simulation	1	^65^
Spatiotemporal analysis	Space-distributed traffic-enhanced LSTM	2	^62^ ^,^ ^66^
	3-Layer spatiotemporal GCN	1	^28^
	USTGCN	1	^44^
	Space-distributed LSTM	1	^62^
	Symbolic regression with stochastic sequential simulation	1	^65^
	COVID-LSTM	1	^71^

Abbreviations: ColaGNN, cross-location attention-based graph neural network; MK-DNN, multikernel density estimation and deep neural network; SOM, self-organizing map; CHGCN, contrastive learning-based hierarchical graph convolutional neural network; TRI, triplet network; C-MPGCN, contrastive learning-based multi-relational graph convolutional neural network; Ca-eSAIR, Temporal extended susceptible-antibody-infectious-removed model with Cellular Automata; PNN, probabilistic neural network; CovidGNN, spatio-temporal graph neural network; MLP, multilayer perceptron; SIRVC, susceptible-infected-recovered with vaccinations and inter-community interaction; GAT, graph attention network; STAN, spatio-temporal attention network; SIR, susceptible-infected-recovered; DCRNN, diffusion convolutional recurrent neural network; GCN, graph convolutional network; RNN, recurrent neural network; SUIR, sensitive-undiagnosed-infected-removed; DeepCOVIDNet, deep spatio-temporal neural network for COVID-19 forecasting; CNN-RNN, convolutional-recurrent neural network hybrid; LSTM-CA, long short-term memory with cellular automata; LSTM-INLA, long short-term memory with integrated nested laplace approximation; ASTGCN, attention-based spatial-temporal graph convolution network; STSGT, spatial-temporal synchronous graph transformer network; STTN, spatio-temporal transformer network; CNN-GRU, convolutional neural network with gated recurrent unit; COVID-19Net, novel spatiotemporal feature extraction parallel deep neural network; ConvLSTM, convolutional long-short term memory; GoogleGNN, google graph neural network; FGC-COVID, fine-grained population mobility data-based community-level COVID-19 prediction model; LSTNET, long- and short-term time-series network; CNNRNN_Res, convolutional and recurrent residual hybrid network; COVID-LSTM, long short-term memory network for COVID-19 forecasting; Ada-STNet, adaptive spatio-temporal graph neural network; USTGCN, unified spatio-temporal graph convolution network; STSGCN, spatio-temporal synchronous graph convolutional network; SGWT, spectral graph wavelet transform; DASTGN, dynamic adaptive spatio-temporal graph network; T-GCN, temporal graph convolutional network; LsOA, lioness optimal algorithm; IT-GCN, interaction-temporal graph convolutional network; SIRD, susceptible-infected-recovered-deceased; ANN, Artificial neural network; GNN, Graph neural network; STConvS2S, Spatio-temporal convolutional sequence-to-sequence neural network; CNN, convolutional neural network; GATv2, Graph Attention Networks, version 2; GRU, gated recurrent unit; LSTM; MPNN, message passing neural network; STGCN, spatiotemporal graph convolutional neural network.

### Analytic focus, evaluation metrics, and software

The variability in the analysis conducted in the reviewed studies, as depicted in Table 3, can be categorized into 4 main types: anomaly analysis, clustering analysis, descriptive analysis, and predictive analysis. Specifically, 31 studies focused solely on predictive analysis^24^^,^^28^^,^^29^^,^^31^^,^^42–45^^,^^47^^,^^54–63^^,^^67^^,^^69^^,^^71–73^; 3 studies solely on descriptive analysis^25^^,^^30^^,^^70^; 3 studies on both predictive and descriptive analyses^26^^,^^32^^,^^53^; 2 studies focused solely on clustering analysis^27^^,^^64^; 2 studies focused solely on anomaly analysis^33^^,^^65^; and 1 study focused on both clustering and predictive analyses.^46^

**Figure 5:**
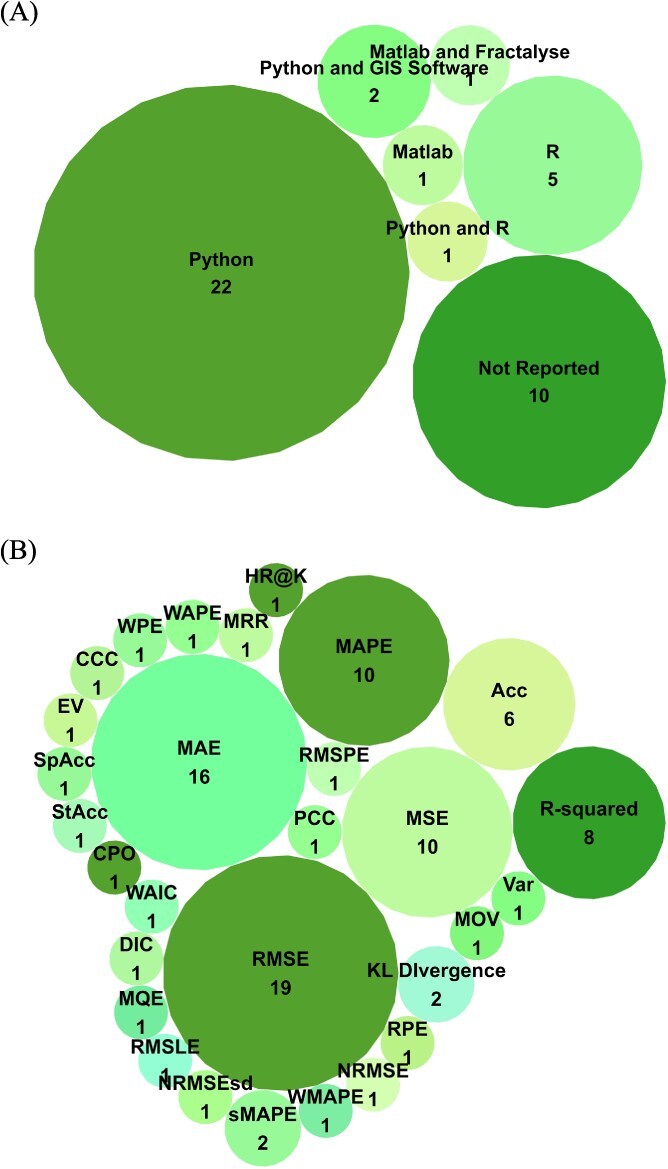
Software and programming languages (A) and evaluation metrics (B) identified.

The software used across the reviewed studies varied significantly, with Python being identified as the most often-used software (Figure 5A). Twenty-two studies used Python only^24^^,^^26^^,^^31–33^^,^^42–44^^,^^47^^,^^48^^,^^53–55^^,^^58^^,^^60^^,^^61^^,^^63^^,^^66–68^^,^^71^^,^^73^; 5 studies used R only^25^^,^^30^^,^^59^^,^^69^^,^^71^; 2 studies used both Python and geographic information system software (QGIS and ArcGIS)^51^^,^^52^; 1 study used both Python and R^57^; 1 study used Matlab only^64^; and 1 study used both Matlab and Fractalyse software.^46^ Ten studies did not report the software or programming language used.^27–29^^,^^45^^,^^49^^,^^50^^,^^56^^,^^62^^,^^65^^,^^72^

Several metrics were used across the reviewed studies to evaluate the performance of spatial or spatiotemporal ML models (Figure 5B). Root mean square error was used in 19 studies^24^^,^^25^^,^^28^^,^^31^^,^^32^^,^^42–44^^,^^47^^,^^49^^,^^50^^,^^55^^,^^57^^,^^59–63^^,^^71^; mean absolute error in 16 studies^25^^,^^28^^,^^29^^,^^42–44^^,^^47^^,^^51^^,^^54–56^^,^^58^^,^^59^^,^^61^^,^^70^^,^^71^; and mean absolute percentage error (MAPE)^26^^,^^28^^,^^42^^,^^43^^,^^45^^,^^58^^,^^61^^,^^62^^,^^66^^,^^71^ and mean square error (MSE)^26^^,^^28^^,^^42^^,^^43^^,^^45^^,^^58^^,^^61^^,^^62^^,^^66^^,^^71^ in 10 studies each. The coefficient of determination (*R*^2^) was used in 8 studies,^24^^,^^25^^,^^30^^,^^43^^,^^49^^,^^65^^,^^67^^,^^70^ accuracy (Acc) in 6 studies,^33^^,^^43^^,^^46^^,^^53^^,^^68^^,^^72^ and Kullback–Leibler divergence,^26^^,^^45^ explained variance score,^43^^,^^63^ and symmetric MAPE^31^^,^^51^ were used in 2 studies each. Additionally, relative prediction error^48^; normalized RMSE^49^; normalized RMSE by SD^51^; mean observed value^59^; Pearson correlation coefficient^73^; concordance correlation coefficient^54^; root mean squared percentage error^28^; weighted MAPE^55^; root mean squared logarithmic error^47^; mean quantization error^64^; deviance information criterion^57^; Watanabe-Akaike information criterion^57^; conditional predictive ordinate^57^; statistical Acc^52^; spatial Acc^52^; mean reciprocal rank^72^; hit ratio at K^72^; weighted prediction error^69^; and weighted absolute prediction error^69^ featured in 1 study each (Figure 5B).

## Discussion

The COVID-19 pandemic has had a profound impact on global society,^7^^,^^74^^,^^75^ driving the need for innovative tools to understand and mitigate its spread. Spatial and spatiotemporal models have played a critical role in this effort.^6^ However, refining these analytical approaches remains essential to effectively inform decision-making and public health guidelines.^9^^,^^10^ Here, we systematically reviewed the spatial and spatiotemporal ML models and context-specific local-level drivers used to study COVID-19 dynamics worldwide. This review highlights critical knowledge gaps for future research, enhances understanding of the spatial and spatiotemporal aspects of COVID-19, and provides insights to inform effective future public health strategies.

The use of spatial and spatiotemporal ML models in analyzing disease dynamics is growing rapidly. This review shows that these models are more commonly applied at broader global scales than in localized contexts, likely due to their computational efficiency. Despite their potential, global models only present an overall estimate for all locations in space.^76^ This limitation is particularly important when considering the variability of disease patterns across different locations, where local nuances are often critical for effective intervention. In contrast, local models offer an alternative by producing estimates for each location in space,^76^ enabling the detection of localized patterns of diseases across space—an aspect of great importance in spatial epidemiology.^77^ Although our review highlights the GRF and GWRF model as the only approaches used in a few studies,^25^^,^^30^^,^^70^ its applicability to spatiotemporal analysis is constrained by challenges in addressing temporal variations. These limitations underscore the need to advance and expand the use of local spatial and spatiotemporal ML models to improve our understanding of COVID-19 dynamics and support more targeted and effective public health interventions.

Understanding the factors influencing the spatial and spatiotemporal dynamics of COVID-19 is crucial for effective disease modeling and public health interventions.^78^^,^^79^ We identified several local-level drivers, including demographic, socioeconomic, and environmental domains, widely used to characterize COVID-19 patterns worldwide. Demographic factors, such as population density, age distribution, and sex, have been extensively explored alongside socioeconomic factors, including income, education, and occupation. Environmental factors such as temperature, air quality, and humidity have also been frequently used. This finding is consistent with the findings of the review conducted by Nazia et al.,^7^ who similarly highlighted the significance of these local-level drivers in understanding COVID-19 dynamics. The consistent identification of these drivers in the literature reflects their long-standing importance in shaping infectious disease dynamics, particularly in determining the susceptibility, spread, and severity of infections across different populations and environments.^80-83^

Recent advances in data integration have significantly enhanced our ability to capture the complex dynamics of COVID-19 by incorporating more dynamic and behavioral dimensions such as mobility and government policy. Mobility data, such as migration/movement indices and travel flows, have been increasingly used to track population movement and assess its role in facilitating viral transmission across geographic areas.^43^^,^^52^^,^^56^^,^^58^^,^^71^^,^^72^ These data often derive from mobile phone records, social media platforms, and aggregated transportation data sets, enabling fine-grained insights into human mobility during different phases of the pandemic. Similarly, government policy data, including the stay-put index and social distancing metrics, reflect the intensity and public compliance with interventions such as lockdowns, travel restrictions, and workplace closures.^48^^,^^50^^,^^53^^,^^55^^,^^71^ The integration of such data has proven critical for modeling the spatial spread and temporal surges of COVID-19, because they capture not only structural vulnerabilities but also behavioral and policy-induced changes in contact rates and exposure risks.^84-86^ These developments highlight a shift toward more responsive and context-aware modeling approaches that move beyond static factors to include real-time, dynamic factors that influence disease propagation. Furthermore, this review shows that most studies have relied on standalone factors to analyze the dynamics of COVID-19, often overlooking the potential of composite indicators, which integrate multiple factors into a single comprehensive score. Although standalone factors provide valuable insights, models with fewer covariates are generally more interpretable and accurate.^87^ Composite indicators can help reduce model complexity by reducing the number of covariates in a model, enhancing performance and reliability.^88^^,^^89^ In many studies, the underutilization of composite indicators represents an opportunity to improve models, offering a more robust framework for understanding COVID-19 dynamics across different contexts.

Few studies have applied spatial and spatiotemporal ML models to analyze COVID-19 dynamics in regions such as Africa, Asia, Australia, and South America. Similarly, it has been highlighted that advanced modeling techniques have been applied sparingly in these regions, which face unique challenges and disease patterns distinct from those in Europe and North America, where research efforts have predominantly focused.^8^ This persistent gap highlights the underrepresentation of these regions in localized COVID-19 dynamics research, which may potentially limit the development of targeted, context-specific, public health interventions. Future studies should focus on these regions, integrating local-level drivers to create more effective, region-specific public health strategies.

Beyond COVID-19, the findings of this review have important implications for ongoing surveillance of other infectious diseases, particularly respiratory illnesses such as influenza, respiratory syncytial virus, and emerging airborne pathogens. Some spatial and spatiotemporal ML models identified in this review can be adapted to capture localized transmission patterns, forecast outbreaks, and integrate diverse contextual drivers relevant to these diseases. Incorporating such models into routine surveillance frameworks could enhance early warning capabilities and support timely, data-driven public health responses across a range of respiratory disease threats.

### Limitations

We acknowledge the following limitations in this study. First, although a rigorous process was used for study selection and data extraction, the possibility of excluding relevant studies cannot be entirely ruled out. Second, the focus on peer-reviewed, English-language journal articles may have resulted in the omission of valuable research published in other languages. Third, information bias may have occurred due to the potential limitations of the databases used. Fourth, inconsistencies in the categorization of models and local-level drivers were observed in some studies but were not further explored. Fifth, variations in models arising from ablation studies were not considered. Sixth, this review prioritized the frequency of local-level drivers’ use rather than their specific impact on COVID-19 dynamics. Seventh, the predefined contextual framework may have obscured potentially significant local-level drivers in alternative contexts.

Additionally, although we report the publication year of the included articles to understand methodological trends over time, we recognize that the time frame of data analyzed is also critical for interpreting results in the context of pandemic phases. Due to inconsistent reporting across studies, we were unable to extract this information systematically. Future reviews could address this by focusing on or encouraging more detailed reporting of data periods. Finally, the emphasis on model prevalence over individual strengths may limit a comprehensive understanding of their capabilities.

## Conclusion

This review comprehensively synthesizes the spatial and spatiotemporal ML models and context-specific, local-level drivers used in COVID-19 research. It provides fresh reflection on and facilitates the improvement of approaches for COVID-19 research. The predominant reliance on global ML models and standalone local-level drivers hinders a granular understanding of COVID-19 dynamics. Hence, we conclude that research should prioritize developing and applying local spatial and spatiotemporal ML models and exploring the potential of composite indicators, especially in geographic areas other than Europe and North America. These advancements are crucial for effective preparedness in the event of future health crises.

## Acknowledgments

H.K.A. is supported by an Australian Government Research Training Program Scholarship.

## Supplementary Material

Web_Material_mxaf017

## Data Availability

This study is a systematic review and does not involve the generation or analysis of primary data. All data used in this review were obtained from published literature cited in the manuscript.
