# Supplementary material for: Spatial and spatiotemporal machine learning models for COVID-19 dynamics: a review of methodology and reporting practices
Source: Epidemiol Rev. 2025 Oct 23;47(1):mxaf017. doi: 10.1093/epirev/mxaf017 (PMC12699224; doi:10.1093/epirev/mxaf017)
Supplement: Web_Material_mxaf017 [file web_material_mxaf017.docx]

**Supplementary materials to**

**Spatial and spatiotemporal machine learning methods for studying COVID-19 dynamics: A methodological review**

**Hassan K. Ajulo^1^, Faith O. Alele^1,2^, Theophilus I. Emeto^1^, Oyelola A. Adegboye^1,3^**

^1^Public Health and Tropical Medicine, James Cook University, Townsville,

QLD, Australia

^2^School of Health, University of the Sunshine Coast, Sippy Downs, QLD, Australia

^3^Menzies School of Health Research, Charles Darwin University, Darwin, NT, Australia

**Table S1:** Details of the used appraisal tool in this study.

**Table S2.** Quality appraisal results.

**Table S3.** List of some nomenclature used in this study.

**Table S1. Details of the used appraisal tool in this study.**

| Question | Consideration | Grade |
| --- | --- | --- |
| Does the paper clearly address aims and objectives? | Is the paper relevant to the objectives of the systematic review of spatial and spatiotemporal machine learning modelling of COVID-19 outcomes? | 0 - not stated  1 - stated but vague  2 - stated and focused |
| Is the setting and population clearly defined? | Does the paper clearly state the setting and population (e.g., number of geographical locations, number of COVID-19 outcome(s), study area)? | 0 - not stated  1 - stated but vague  2 - stated and focused |
| Is the model structure clearly described and appropriate for the research question? | Is there a description of model structure/architecture? Does the model structure/architecture include local-level drivers within the predefined contexts? | 0 - not appropriate model structure/architecture or no description of model and no local-level drivers within the predefined contexts  1 - incomplete  2 - complete |
| Are the modelling methods appropriate for the research question? | Are the modelling methods clearly described, and suited to the research questions? | 0 - not appropriate modelling method or no description of model  1 - incomplete description  2 - complete description |
| Are the parameters, ranges and data source specified? | Are hyperparameters and their values reported? Are data sources reported? | 0 - poorly reported  1 - some information missing  2 - complete reporting of hyperparameters and data sources |
| Is the quality of data considered? | Are data limitations clearly reported? | 0 - not reported  1 - not clearly reported  2 - clearly reported |
| Have the results been clearly and completely presented? | Do the results match the aims and objectives? | 0 - not reported, very unclear  1 - stated, but not directly aligned with research question  2 - valuable and aligned with research question |
| Are the results appropriately interpreted and discussed in context? | Are the results of the study discussed in context and generalisability considered? Are study limitations discussed? | 0 - no discussion  1 - some discussion but key points and/or limitations missed  2 - full discussion of key points, limitations discussed |

**Table S2. Quality appraisal results**

| ID | Title | Reference | Q1 | Q2 | Q3 | Q4 | Q5 | Q6 | Q7 | Q8 | Total score | Rating |
| --- | --- | --- | --- | --- | --- | --- | --- | --- | --- | --- | --- | --- |
| 1 | Deep learning of contagion dynamics on complex networks | ^1^ | 2 | 2 | 2 | 2 | 2 | 1 | 2 | 2 | 15 | Very High |
| 2 | A graph convolutional network for predicting COVID-19 dynamics in 190 regions/countries | ^2^ | 2 | 2 | 1 | 2 | 2 | 1 | 2 | 1 | 13 | High |
| 3 | Interaction-Temporal GCN: A Hybrid Deep Framework for Covid-19 Pandemic Analysis | ^3^ | 2 | 2 | 1 | 2 | 2 | 0 | 2 | 1 | 12 | High |
| 4 | Predicting COVID-19 using lioness optimisation algorithm and graph convolution network | ^4^ | 2 | 2 | 1 | 2 | 1 | 1 | 2 | 1 | 12 | High |
| 5 | Dynamic adaptive spatio-temporal graph network for COVID-19 forecasting | ^5^ | 2 | 2 | 1 | 2 | 1 | 1 | 2 | 2 | 13 | High |
| 6 | A deep learning approach for Spatio-Temporal forecasting of new cases and new hospital admissions of COVID-19 spread in Reggio Emilia, Northern Italy | ^6^ | 2 | 2 | 2 | 2 | 2 | 2 | 2 | 2 | 16 | Very High |
| 7 | A Multivariate Spatiotemporal Model of COVID-19 Epidemic Using Ensemble of ConvLSTM Networks | ^7^ | 2 | 2 | 1 | 2 | 2 | 0 | 1 | 1 | 11 | High |
| 8 | A new RNN based machine learning model to forecast COVID-19 incidence, enhanced by the use of mobility data from the bike-sharing service in Madrid | ^8^ | 2 | 2 | 1 | 2 | 2 | 2 | 2 | 2 | 15 | Very High |
| 9 | A spatiotemporal machine learning approach to forecasting COVID-19 incidence at the county level in the USA | ^9^ | 2 | 2 | 2 | 2 | 2 | 1 | 2 | 2 | 15 | Very High |
| 10 | A Stochastic Model of an Early Warning System for Detecting Anomalous Incidence Values of COVID-19 | ^10^ | 2 | 2 | 1 | 2 | 2 | 1 | 2 | 2 | 14 | Very High |
| 11 | An Epidemiological Neural network exploiting Dynamic Graph Structured Data applied to the COVID-19 outbreak | ^11^ | 2 | 2 | 2 | 2 | 2 | 2 | 2 | 2 | 16 | Very High |
| 12 | Analysis of Spatial Spread Relationships of Coronavirus (COVID-19) Pandemic in the World using Self Organizing Maps | ^12^ | 2 | 2 | 1 | 2 | 2 | 0 | 2 | 2 | 13 | High |
| 13 | Analysis of the Spatio-Temporal Dynamics of COVID-19 in Massachusetts via Spectral Graph Wavelet Theory | ^13^ | 2 | 2 | 1 | 2 | 2 | 1 | 2 | 1 | 13 | High |
| 14 | Borough-level COVID-19 forecasting in London using deep learning techniques and a novel MSE-Moran’s I loss function | ^14^ | 2 | 2 | 2 | 2 | 2 | 2 | 2 | 2 | 16 | Very High |
| 15 | Combining graph neural networks and spatio-temporal disease models to improve the prediction of weekly COVID-19 cases in Germany | ^15^ | 2 | 2 | 2 | 2 | 2 | 2 | 2 | 2 | 16 | Very High |
| 16 | Explaining Causal Influence of External Factors on Incidence Rate of Covid-19 | ^16^ | 2 | 2 | 2 | 2 | 2 | 1 | 2 | 1 | 14 | Very High |
| 17 | Fine-Grained Population Mobility Data-Based Community-Level COVID-19 Prediction Model | ^17^ | 2 | 2 | 2 | 2 | 2 | 1 | 2 | 2 | 15 | Very High |
| 18 | Forecasting the COVID-19 Space-Time Dynamics in Brazil with Convolutional Graph Neural Networks and Transport Modals | ^18^ | 2 | 2 | 2 | 2 | 2 | 2 | 2 | 2 | 16 | Very High |
| 19 | Learning where to look for COVID-19 growth: Multivariate analysis of COVID-19 cases over time using explainable convolution-LSTM | ^19^ | 2 | 2 | 2 | 2 | 2 | 1 | 2 | 2 | 15 | Very High |
| 20 | Modeling the Geospatial Evolution of COVID-19 using Spatio-temporal Convolutional Sequence-to-sequence Neural Networks | ^20^ | 2 | 1 | 2 | 2 | 2 | 2 | 2 | 2 | 15 | Very High |
| 21 | Novel spatiotemporal feature extraction parallel deep neural network for forecasting confirmed cases of coronavirus disease 2019 | ^21^ | 2 | 1 | 2 | 2 | 2 | 1 | 2 | 2 | 14 | Very High |
| 22 | Predicting COVID-19 positivity and hospitalisation with multi-scale graph neural networks | ^22^ | 2 | 2 | 2 | 2 | 2 | 2 | 2 | 2 | 16 | Very High |
| 23 | Spatial and Temporal Spread of the COVID-19 Pandemic Using Self Organising Neural Networks and a Fuzzy Fractal Approach | ^23^ | 2 | 2 | 1 | 2 | 2 | 0 | 2 | 1 | 12 | High |
| 24 | Spatial-Temporal Synchronous Graph Transformer network (STSGT) for COVID-19 forecasting | ^24^ | 2 | 2 | 1 | 2 | 2 | 1 | 2 | 1 | 13 | High |
| 25 | Spatiotemporal Modeling of the Association between Neighborhood Factors and COVID-19 Incidence Rates in Scotland | ^25^ | 2 | 2 | 2 | 2 | 1 | 2 | 2 | 2 | 15 | Very High |
| 26 | The Spread of the COVID-19 Outbreak in Brazil: An Overview by Kohonen Self-Organizing Map Networks | ^26^ | 2 | 1 | 1 | 2 | 2 | 2 | 2 | 2 | 14 | Very High |
| 27 | Toward Combatting COVID-19: A Risk Assessment System | ^27^ | 2 | 2 | 1 | 2 | 2 | 2 | 2 | 1 | 14 | Very High |
| 28 | A Bayesian machine learning approach for spatio-temporal prediction of COVID-19 cases | ^28^ | 2 | 2 | 2 | 2 | 2 | 2 | 2 | 2 | 16 | Very High |
| 29 | A new method for spatio-temporal transmission prediction of COVID-19 | ^29^ | 2 | 2 | 2 | 2 | 2 | 2 | 2 | 2 | 16 | Very High |
| 30 | Deep Spatiotemporal Model for COVID-19 Forecasting | ^30^ | 2 | 2 | 1 | 2 | 2 | 1 | 2 | 2 | 14 | Very High |
| 31 | DeepCOVIDNet: An Interpretable Deep Learning Model for Predictive Surveillance of COVID-19 Using Heterogeneous Features and Their Interactions | ^31^ | 2 | 2 | 2 | 2 | 2 | 2 | 2 | 2 | 16 | Very High |
| 32 | Distribution of the environmental and socioeconomic risk factors on COVID-19 death rate across continental USA: a spatial non-linear analysis | ^32^ | 2 | 2 | 2 | 2 | 2 | 2 | 2 | 2 | 16 | Very High |
| 33 | Ranking the importance of demographic, socioeconomic, and underlying health factors on US COVID-19 deaths: A geographical random forest approach | ^33^ | 2 | 2 | 2 | 2 | 2 | 2 | 2 | 2 | 16 | Very High |
| 34 | Machine learning spatio-temporal epidemiological model to evaluate Germany-county-level COVID-19 risk | ^34^ | 2 | 2 | 1 | 2 | 2 | 2 | 2 | 2 | 15 | Very High |
| 35 | STAN: spatio-temporal attention network for pandemic prediction using real-world evidence | ^35^ | 2 | 2 | 2 | 2 | 2 | 2 | 2 | 2 | 16 | Very High |
| 36 | Understanding and predicting the spatio-temporal spread of COVID-19 via integrating diffusive graph embedding and compartmental models | ^36^ | 2 | 1 | 2 | 2 | 2 | 2 | 2 | 2 | 15 | Very High |
| 37 | Using a Layered Ensemble of Physics-Guided Graph Attention Networks to Predict COVID-19 Trends | ^37^ | 2 | 2 | 2 | 2 | 2 | 2 | 2 | 2 | 16 | Very High |
| 38 | A probabilistic spatio-temporal neural network to forecast COVID-19 counts | ^38^ | 2 | 2 | 1 | 2 | 1 | 2 | 2 | 2 | 14 | Very High |
| 39 | A spatiotemporal epidemiological prediction model to inform county-level covid-19 risk in the United States | ^39^ | 2 | 2 | 1 | 2 | 2 | 2 | 2 | 2 | 15 | Very High |
| 40 | Space-Distributed Traffic-Enhanced LSTM-Based Machine Learning Model for COVID-19 Incidence Forecasting | ^40^ | 2 | 2 | 1 | 2 | 2 | 2 | 2 | 2 | 15 | Very High |
| 41 | Forecasting infections with spatio-temporal graph neural networks: a case study of the Dutch SARS-CoV-2 spread | ^41^ | 2 | 2 | 2 | 2 | 2 | 2 | 2 | 2 | 16 | Very High |
| 42 | Predicting the transmission trend of respiratory viruses in new regions via geospatial similarity learning | ^42^ | 2 | 2 | 2 | 2 | 1 | 2 | 2 | 2 | 15 | Very High |
| Mean Score | |  |  | | | | | | | | 14.62 | Very High |
| Median Score | |  |  | | | | | | | | 15 | Very High |

Table S3. List of some nomenclature used in this study.

| S/N | Nomenclature | Referred to |
| --- | --- | --- |
| 1 | STGCN | Spatiotemporal graph convolutional networks |
| 2 | GCN | Graph convolutional networks |
| 3 | CovidGNN | Spatio-temporal graph neural network |
| 4 | LsOA-GCN | Lioness optimal algorithm and graph convolution network |
| 5 | T-GCN | Temporal graph convolutional network |
| 6 | GRF | Geographically random forest |
| 7 | GAT | Graph attention network |
| 8 | LSTM | Long-short term memory |
| 9 | ConvLSTM | Convolutional long-short term memory |
| 10 | CNN | Convolutional neural network |
| 11 | DASTGN | Dynamic adaptive spatio-temporal graph network |
| 12 | ColaGNN | Cross-location attention-based graph neural networks |
| 13 | STSGCN | Spatio-temporal synchronous graph convolutional networks |
| 14 | GWNet | Graph wavenet |
| 15 | USTGCN | Unified spatio-temporal graph convolution network |
| 16 | Ada-STNet | Adaptive spatio-temporal graph neural network |
| 17 | SOM | Self-organizing map |
| 18 | SGWT | Spectral graph wavelet transform |
| 19 | FGC-COVID | Fine-grained population mobility data-based community-level covid-19 prediction model |
| 20 | LSTNET | Long- and short-term time-series network |
| 21 | DCRNN | Diffusion convolutional recurrent neural network |
| 22 | Google-GNN | Google graph neural network |
| 23 | GNN | Graph neural network |
| 24 | STConvS2S | Spatio-temporal convolutional sequence-to-sequence neural networks |
| 25 | MPNN | Message passing neural network |
| 26 | STSGT | Spatial-temporal synchronous graph transformer network |
| 27 | ASTGCN | Attention-based spatial-temporal graph convolution network |
| 28 | STAN | Spatio-temporal attention network |
| 29 | CA | Cellular automata |
| 30 | SUIR | Sensitive-undiagnosed-infected-removed |
| 31 | SIR | Susceptible-infected-recovered |
| 32 | SIRD | Susceptible-infected-recovered-deceased |
| 33 | PCC | Pearson correlation coefficient |
| 34 | MSE | Mean square error |
| 35 | RMSE | Root mean square error |
| 36 | MAE | Mean absolute error |
| 37 | RMSPE | Root mean squared percentage error |
| 38 | MAPE | Mean absolute percentage error |
| 39 | Acc | Accuracy |
| 40 | Var/EV | Explained variance score |
| 41 | R-squared | Coefficient of determination |
| 42 | KL Divergence | Kullback–Liebler divergence |
| 43 | MILoss | Moran’s I loss |
| 44 | RPE | Relative prediction error |
| 45 | sMAPE | Symmetric mean absolute percentage error |
| 46 | WMAPE | Weighted mean absolute percentage error |
| 47 | NRMSEsd | Normalized root mean square error by the standard deviation |
| 48 | RMSLE | Root mean squared logarithmic error |
| 49 | MQE | Mean quantization error |
| 50 | DIC | Deviance information criterion |
| 51 | WAIC | Watanabe Akaike information criterion |
| 52 | CPO | Conditional predictive ordinate |
| 53 | StAcc | Statistical accuracy |
| 54 | SpAcc | Spatial accuracy |
| 55 | CCC | Concordance correlation coefficient |
| 56 | MLP | Multilayer perceptron |
| 57 | ARE | Average relative error |
| 58 | MOV | Mean observed value |
| 59 | ANN | Artificial neural network |
| 60 | COVID-19Net | Novel spatiotemporal feature extraction parallel deep neural network |
| 61 | MK-DNN | Multikernel density estimation-deep neural network |
| 62 | SIRVC | Susceptible-infected-recovered with vaccinations and inter-community interaction |
| 63 | eSAIR | Temporal extended susceptible-antibody-infectious-removed |
| 64 | CHGCN | Contrastive learning-based hierarchical graph convolutional neural network |
| 65 | TRI | Triplet network |
| 66 | C-MPGCN | Contrastive learning-based multi-relational graph convolutional neural network |
| 67 | MRR | A ranking-based evaluation metric (1/rank) |
| 68 | HR@K | The probability that a model returns the ground-truth region 𝑅 within the top-𝑘 results among a model’s returned list (ranked according to the similarity score) for a query region 𝑄 |
| 69 | WPE | Weighted prediction error |
| 70 | WAPE | Weighted absolute prediction error |
| 71 | NRMSE | Normalized root mean square error |

**Reference**

1. Murphy C, Laurence E and Allard A. Deep learning of contagion dynamics on complex networks. *Nature Communications* 2021; 12: 4720. DOI: 10.1038/s41467-021-24732-2.

2. Anno S, Hirakawa T, Sugita S, et al. A graph convolutional network for predicting COVID-19 dynamics in 190 regions/countries. *Front Public Health* 2022; 10: 911336. 20220803. DOI: 10.3389/fpubh.2022.911336.

3. Yu Z, Zheng X, Yang Z, et al. Interaction-Temporal GCN: A Hybrid Deep Framework For Covid-19 Pandemic Analysis. *IEEE Open J Eng Med Biol* 2021; 2: 97-103. 20210304. DOI: 10.1109/ojemb.2021.3063890.

4. Li D, Ren X and Su Y. Predicting COVID-19 using lioness optimization algorithm and graph convolution network. *Soft comput* 2023; 27: 5437-5501. 20230109. DOI: 10.1007/s00500-022-07778-2.

5. Pu X, Zhu J, Wu Y, et al. Dynamic adaptive spatioâ€“temporal graph network for COVID-19 forecasting. *CAAI Transactions on Intelligence Technology* 2023. DOI: doi:10.1049/cit2.12238.

6. Sciannameo V, Goffi A, Maffeis G, et al. A deep learning approach for Spatio-Temporal forecasting of new cases and new hospital admissions of COVID-19 spread in Reggio Emilia, Northern Italy. *Journal of Biomedical Informatics* 2022; 132. DOI: doi:10.1016/j.jbi.2022.104132.

7. Paul SK, Jana S and Bhaumik P. A Multivariate Spatiotemporal Model of COVID-19 Epidemic Using Ensemble of ConvLSTM Networks. *Journal of The Institution of Engineers (India): Series B* 2020. DOI: doi:10.1007/s40031-020-00517-x.

8. Muñoz-Organero M, Callejo P and Hombrados-Herrera M. A new RNN based machine learning model to forecast COVID-19 incidence, enhanced by the use of mobility data from the bike-sharing service in Madrid. *Heliyon* 2023; 9: e17625. 20230624. DOI: 10.1016/j.heliyon.2023.e17625.

9. Lucas B, Vahedi B and Karimzadeh M. A spatiotemporal machine learning approach to forecasting COVID-19 incidence at the county level in the USA. *Int J Data Sci Anal* 2023; 15: 247-266. 20220115. DOI: 10.1007/s41060-021-00295-9.

10. Duarte A, Soares A, Pereira M, et al. A Stochastic Model of an Early Warning System for Detecting Anomalous Incidence Values of COVID-19. *MATHEMATICAL GEOSCIENCES* 2023. DOI: doi:10.1007/s11004-023-10096-4.

11. La Gatta V, Moscato V, Postiglione M, et al. An Epidemiological Neural network exploiting Dynamic Graph Structured Data applied to the COVID-19 outbreak. *IEEE Transactions on Big Data* 2020. DOI: doi:10.1109/TBDATA.2020.3032755.

12. Melin P, Monica J, Sanchez D, et al. Analysis of Spatial Spread Relationships of Coronavirus (COVID-19) Pandemic in the World using Self Organizing Maps. *CHAOS SOLITONS & FRACTALS* 2020; 138. DOI: doi:10.1016/j.chaos.2020.109917 WE - Science Citation Index Expanded (SCI-EXPANDED).

13. Geng R, Gao Y, Zhang H, et al. Analysis of the Spatio-Temporal Dynamics of COVID-19 in Massachusetts via Spectral Graph Wavelet Theory. *IEEE TRANSACTIONS ON SIGNAL AND INFORMATION PROCESSING OVER NETWORKS* 2022; 8. DOI: doi:10.1109/TSIPN.2022.3193252 WE - Science Citation Index Expanded (SCI-EXPANDED).

14. Olsen F, Schillaci C, Ibrahim M, et al. Borough-level COVID-19 forecasting in London using deep learning techniques and a novel MSE-Moran's I loss function. *Results Phys* 2022; 35: 105374. 20220224. DOI: 10.1016/j.rinp.2022.105374.

15. Fritz C, Dorigatti E and Rügamer D. Combining graph neural networks and spatio-temporal disease models to improve the prediction of weekly COVID-19 cases in Germany. *Sci Rep* 2022; 12: 3930. 20220310. DOI: 10.1038/s41598-022-07757-5.

16. Paul SK, Jana S and Bhaumik P. Explaining Causal Influence of External Factors on Incidence Rate of Covid-19. *SN Computer Science* 2021; 2. DOI: doi:10.1007/s42979-021-00864-6.

17. Jia P, Chen L and Lyu D. Fine-Grained Population Mobility Data-Based Community-Level COVID-19 Prediction Model. *Cybernetics and Systems* 2022. DOI: doi:10.1080/01969722.2022.2103614.

18. Oliveira L, Oliva J, Ribeiro M, et al. Forecasting the COVID-19 Space-Time Dynamics in Brazil With Convolutional Graph Neural Networks and Transport Modals. *IEEE ACCESS* 2022; 10. DOI: doi:10.1109/ACCESS.2022.3195535 WE - Science Citation Index Expanded (SCI-EXPANDED).

19. Yudistira N, Sumitro S, Nahas A, et al. Learning where to look for COVID-19 growth: Multivariate analysis of COVID-19 cases over time using explainable convolution-LSTM. *APPLIED SOFT COMPUTING* 2021; 109. DOI: doi:10.1016/j.asoc.2021.107469.

20. Cardoso M, Cavalheiro A, Borges A, et al. Modeling the Geospatial Evolution of COVID-19 using Spatio-temporal Convolutional Sequence-to-sequence Neural Networks. *ACM TRANSACTIONS ON SPATIAL ALGORITHMS AND SYSTEMS* 2022; 8. DOI: doi:10.1145/3550272 WE - Emerging Sources Citation Index (ESCI).

21. Huang CJ, Shen Y, Kuo PH, et al. Novel spatiotemporal feature extraction parallel deep neural network for forecasting confirmed cases of coronavirus disease 2019. *Socioecon Plann Sci* 2022; 80: 100976. 20201125. DOI: 10.1016/j.seps.2020.100976.

22. Skianis K, Nikolentzos G, Gallix B, et al. Predicting COVID-19 positivity and hospitalization with multi-scale graph neural networks. *Sci Rep* 2023; 13: 5235. 20230331. DOI: 10.1038/s41598-023-31222-6.

23. Melin P, Castillo, Oscar. Spatial and Temporal Spread of the COVID-19 Pandemic Using Self Organizing Neural Networks and a Fuzzy Fractal Approach. *Sustainability* 2021; 13. DOI: doi:10.3390/su13158295.

24. Banerjee S, Dong M and Shi W. Spatialâ€“Temporal Synchronous Graph Transformer network (STSGT) for COVID-19 forecasting. *Smart Health* 2022; 26. DOI: doi:10.1016/j.smhl.2022.100348.

25. Wang R, Clemens T, Douglas M, et al. Spatiotemporal Modeling of the Association between Neighborhood Factors and COVID-19 Incidence Rates in Scotland. *The Professional Geographer* 2023; 75: 803-815. DOI: 10.1080/00330124.2023.2194363.

26. Galvan D, Effting L, Cremasco H, et al. The Spread of the COVID-19 Outbreak in Brazil: An Overview by Kohonen Self-Organizing Map Networks. *Medicina (Kaunas)* 2021; 57 20210303. DOI: 10.3390/medicina57030235.

27. Wang Q, Guo Y, Ji T, et al. Toward Combatting COVID-19: A Risk Assessment System. *IEEE Internet Things J* 2021; 8: 15953-15964. 20210331. DOI: 10.1109/jiot.2021.3070042.

28. Niraula P, Mateu J and Chaudhuri S. A Bayesian machine learning approach for spatio-temporal prediction of COVID-19 cases. *Stoch Environ Res Risk Assess* 2022; 36: 2265-2283. 20220125. DOI: 10.1007/s00477-021-02168-w.

29. Wang P, Liu H, Zheng X, et al. A new method for spatio-temporal transmission prediction of COVID-19. *Chaos Solitons Fractals* 2023; 167: 112996. 20221227. DOI: 10.1016/j.chaos.2022.112996.

30. Muñoz-Organero M and Queipo-Álvarez P. Deep Spatiotemporal Model for COVID-19 Forecasting. *Sensors (Basel)* 2022; 22 20220505. DOI: 10.3390/s22093519.

31. Ramchandani A, Fan C and Mostafavi A. DeepCOVIDNet: An Interpretable Deep Learning Model for Predictive Surveillance of COVID-19 Using Heterogeneous Features and Their Interactions. *IEEE Access* 2020; 8: 159915-159930. 20200828. DOI: 10.1109/access.2020.3019989.

32. Luo Y, Yan J and McClure S. Distribution of the environmental and socioeconomic risk factors on COVID-19 death rate across continental USA: a spatial nonlinear analysis. *Environ Sci Pollut Res Int* 2021; 28: 6587-6599. 20201001. DOI: 10.1007/s11356-020-10962-2.

33. Grekousis G, Feng Z, Marakakis I, et al. Ranking the importance of demographic, socioeconomic, and underlying health factors on US COVID-19 deaths: A geographical random forest approach. *Health Place* 2022; 74: 102744. 20220131. DOI: 10.1016/j.healthplace.2022.102744.

34. Wang L, Xu T, Stoecker T, et al. Machine learning spatio-temporal epidemiological model to evaluate Germany-county-level COVID-19 risk. *Machine Learning: Science and Technology* 2021; 2: 035031. DOI: 10.1088/2632-2153/ac0314.

35. Gao J, Sharma R, Qian C, et al. STAN: spatio-temporal attention network for pandemic prediction using real-world evidence. *J Am Med Inform Assoc* 2021; 28: 733-743. DOI: 10.1093/jamia/ocaa322.

36. Zhang T and Li J. Understanding and predicting the spatio-temporal spread of COVID-19 via integrating diffusive graph embedding and compartmental models. *Trans GIS* 2021; 25: 3025-3047. 20210716. DOI: 10.1111/tgis.12803.

37. Sun C, Kumarasamy VK, Liang Y, et al. Using a Layered Ensemble of Physics-Guided Graph Attention Networks to Predict COVID-19 Trends. *Applied Artificial Intelligence* 2022; 36: 2055989. DOI: 10.1080/08839514.2022.2055989.

38. Ravenda F, Cesarini M, Peluso S, et al. A probabilistic spatio-temporal neural network to forecast COVID-19 counts. *International Journal of Data Science and Analytics* 2024. DOI: 10.1007/s41060-024-00525-w.

39. Zhou Y, Wang, L., Zhang, L., Shi, L., Yang, K., He, J., Bangyao, Z., Overton, W., Purkayastha, S., & Song, P. A Spatiotemporal Epidemiological Prediction Model to Inform County-Level COVID-19 Risk in the United States. *Harvard Data Science Review, (Special Issue 1)* 2020. DOI: <https://doi.org/10.1162/99608f92.79e1f45e>.

40. Muñoz-Organero M. Space-Distributed Traffic-Enhanced LSTM-Based Machine Learning Model for COVID-19 Incidence Forecasting. *Computational Intelligence and Neuroscience* 2022; 2022: 4307708. DOI: <https://doi.org/10.1155/2022/4307708>.

41. Croft VM, van Iersel SCJL and Della Santina C. Forecasting infections with spatio-temporal graph neural networks: a case study of the Dutch SARS-CoV-2 spread. *Frontiers in Physics* 2023; 11. Original Research.

42. Zhao Y, Hu M, Jin Y, et al. Predicting the transmission trend of respiratory viruses in new regions via geospatial similarity learning. *International Journal of Applied Earth Observation and Geoinformation* 2023; 125: 103559. DOI: <https://doi.org/10.1016/j.jag.2023.103559>.
